# The Efficacy of Manual Therapy Approaches on Pain, Maximum Mouth Opening and Disability in Temporomandibular Disorders: A Systematic Review of Randomised Controlled Trials

**DOI:** 10.3390/life13020292

**Published:** 2023-01-20

**Authors:** Leonardo Sette Vieira, Priscylla Ruany Mendes Pestana, Júlio Pascoal Miranda, Luana Aparecida Soares, Fabiana Silva, Marcus Alessandro Alcantara, Vinicius Cunha Oliveira

**Affiliations:** 1 Postgraduate Program in Rehabilitation and Functional Performance, Universidade Federal dos Vales do Jequitinhonha e Mucuri (UFVJM), Diamantina 39100-000, Brazil; 2 Cirklo Health Education, Barão de Ubá, Porto Alegre 90450-090, Brazil; 3 Postgraduate Program in Health Sciences, Universidade Federal dos Vales do Jequitinhonha e Mucuri (UFVJM), Diamantina 39100-000, Brazil

**Keywords:** temporomandibular joint disorders, temporomandibular joint dysfunction syndrome, musculoskeletal manipulations, manual therapies, systematic review

## Abstract

Temporomandibular disorder (TMD) is a common condition disabling people and bringing up costs. The aim of this study was to investigate the effects of manual therapy on pain intensity, maximum mouth opening (MMO) and disability. Searches were conducted in six databases for randomised controlled trials (RCTs). Selection of trials, data extraction and methodological quality assessment were conducted by two reviewers with discrepancies resolved by a third reviewer. Estimates were presented as mean differences (MDs) or standardized mean differences (SMDs) with 95% confidence intervals (CIs). Quality of the evidence was assessed using the GRADE approach. Twenty trials met the eligibility criteria and were included. For pain intensity, high and moderate quality evidence demonstrated the additional effects of manual therapy at short- (95% CI −2.12 to −0.82 points) and long-term (95% CI −2.17 to −0.40 points) on the 0–10 points scale. For MMO, moderate to high quality evidence was found in favour of manual therapy alone (95% CI 0.01 to 7.30 mm) and its additional effects (95% CI 1.58 to 3.58 mm) at short- and long-term (95% CI 1.22 to 8.40 mm). Moderate quality evidence demonstrated an additional effect of manual therapy for disability (95% CI = −0.87 to −0.14). Evidence supports manual therapy as effective for TMD.

## 1. Introduction

Temporomandibular disorders (TMD) can be defined as a group of pathologies of the temporomandibular joint and muscles involved [1]. TMD can be classified as myogenic (i.e., muscle and myofascial origin), arthrogenic, mixed and joint-related disorders (i.e., disc displacements with or without reduction, arthritis or subluxation) according to The Diagnostic Criteria for Temporomandibular Disorders (DC/TMD) [1,2]. It is a common health condition worldwide with an estimated prevalence ranging from 11% to 31%, and is especially high in people with multiple sclerosis [3,4]. After a new episode of TMD, 27% of people persist with significant pain one year later [5,6,7], and recurrence is common [8]. Its related pain and disability bring direct (e.g., use of medication to alleviate symptoms) and indirect (e.g., productivity loss) costs [9,10,11,12,13]; therefore, effective management of the condition is important.

Management options for TMD include occlusal splints, cognitive behavioural therapy, acupuncture, manual therapy, therapeutic exercises, nonsteroidal anti-inflammatory drugs, surgical treatment and others [14,15,16,17,18,19]. Counselling and a conservative approach are generally advocated as a first management choice by health professionals for patients with disabling TMD [19]. Previous systematic review suggested that manual therapy may improve pain intensity, function, and oral health-related quality of life in this population [19]; however, their scope and methods adopted might have compromised the effect estimates presented. These consist of the inclusion of trials that did not adequately compare manual therapy to investigate its effectiveness and the inclusion of non-randomised controlled trials (RCTs) [19]. In addition, the evidence needs updating, as new trials have been published since then. Thus, a new systematic review of randomized controlled trials that methodologically isolate manual therapy to assess its isolate or additional effects is needed to inform the current state of the evidence on this topic.

The aim of this systematic review of RCTs was to investigate the efficacy of manual therapy approaches and whether they enhance effects when combined with other active intervention on pain intensity, maximum mouth opening (MMO) and disability in TMD. The quality of evidence was assessed using the Grading of Recommendations Assessment (GRADE) approach [20].

## 2. Methods

### 2.1. Study Design

This systematic review of RCTs followed the Cochrane recommendations [21] and the Preferred Reporting Items for Systematic Reviews and Meta-Analyses (PRISMA) checklist [22] (Supplementary File S1: PRISMA checklist). Its protocol was prospectively registered in the International Prospective Registry of Systematic Reviews (PROSPERO) platform (CRD42022372298) and Open Science Framework (DOI: 10.17605/OSF.IO/XSN42).

### 2.2. Search Strategy and Study Selection

Searches were conducted on MEDLINE, COCHRANE, EMBASE, AMED, PSYCINFO and PEDRO without language or date restrictions up to 3 October 2022. Search terms were related to “randomised controlled trials” and “temporomandibular disorders”. A detailed search strategy is in the Supplementary File S2: Search Strategy. In addition, we hand searched identified systematic reviews published in the field for potentially relevant full texts that do not identify in the optimized searches. After searches, the retrieved references were exported to an Endnote^®^ file and duplicates were removed. Then, two independent reviewers (JPM and LAS) screened titles and abstracts and assessed potential full texts. Those trials fulfilling our eligibility criteria were included. The between-reviewer discrepancies were resolved by a third reviewer (VCO).

### 2.3. Inclusion and Exclusion Criteria

We included RCTs investigating people of both sexes, regardless of age, diagnosed with TMD of any duration or type/classification, i.e., myogenic, arthrogenic, mixed and joint-related disorders. The intervention of interest was any manual therapy approach, i.e., any clinician-applied movement of the joints and other structures such as joint mobilization or manipulation (thrust), massage, myofascial release techniques/soft-tissue mobilization, muscle energy techniques, passive stretching and others, as investigated previously [17], using the hands and/or any assisting device. We compared the intervention of interest with control (i.e., placebo, no intervention, waiting list or sham) to investigate the potential specific effects of manual therapy. To investigate whether manual therapy approaches enhance the estimated effects of other active intervention, we also considered comparisons between manual therapy approaches combined with any other active intervention and the other active intervention standing alone. Our outcomes of interest were pain intensity, maximum mouth opening/MMO (i.e., maximum distance between the edge of the upper incisors and the edge of the lower incisors with or without pain) and oral disability. We considered any valid instrument such as Visual Analog Scale—VAS or Numerical Rating Scales—NRS [23] for pain intensity, ruler and caliper for maximum mouth opening [24], and Jaw Functional Limitation Scale (JFLS) [25] and Mandibular Function Impairment Questionnaire (MFIQ) [26] for disability.

### 2.4. Data Extraction

Two independent reviewers (JPM and LAS) extracted characteristics and outcome data from included trials. Between-reviewer disagreements were resolved by a third reviewer (VCO). The extracted data include study type; the participants; details about the interventions and comparator; outcomes and time-points for the purpose of this review. For our outcomes of interest, we extracted post-intervention means (first option) or within-group mean changes over time, standard deviations (SDs) and sample sizes for each of our groups of interest to investigate the effects at immediate- short- and long-term. Immediate effects were considered as the point of measure right after a single session of manual therapy. We considered short-term effects follow-ups from one to 12 weeks after randomization and long-term effects as follow-ups over 12 weeks after randomization. If more than one time-point was available within the same follow-up period, the one closer to the end of the intervention was considered. When outcome data were not reported, at first, the authors were contacted. If we received no answer, we imputed when possible following the recommendations [21]. When authors did not respond and imputation was not possible, trials were excluded from the quantitative analysis.

### 2.5. Risk of Bias Assessment

Two independent reviewers (JPM and LAS) assessed the risk of bias of included trials using the 0–10 PEDRO scale [27]. According to this scale, higher scores represent a higher methodological quality. Discrepancies were resolved by a third reviewer (VCO). When available, we used scores already on the PEDRO database (https://pedro.org.au/, accessed on 10 November 2022).

### 2.6. Data Analysis

When possible, data were converted to a common scale and meta-analysis was conducted using random-effects models (DerSimonian and Laird method). Mean differences (MDs) and 95% CIs were reported in forest-plots. When it was not possible to convert data to a common scale, estimates were presented as standardized mean differences (SMDs). The clinical importance of the interventions of interest was interpreted by comparing the estimated effect sizes and 95% CI in association with the minimum clinically important difference (MCID) of the outcome of interest, or Minimal Detectable Change (MDC) when MCID was not available. MCID considered for pain intensity was 2 points on the 0–10 points scale [28]; MDC of 5 mm for MMO [29]; MDC of 8 points on the 0–68 on Migraine Functional Impact Questionnaire (MFIQ) or 7 points on the 0–63 on Craniofacial Pain and Disability Inventory (CF-PDI) [29] for disability. We used the Hedges’ g effect size measure when estimates were presented as SMD, considering the cut-off points of 0.20, 0.50 and 0.80 for small, medium and large effects, respectively. All analyses were performed in the Comprehensive Meta-analysis software, version 2.2.04 (Biostat, Englewood, NJ, USA). Heterogeneity was assessed using I². We planned to perform subgroup and sensitivity analyses to assess the impact of potential sources of heterogeneity and risk of bias on the estimates. All procedures followed the recommended methods [21].

Two independent reviewers (JPM and LAS) assessed the quality of the current evidence using the GRADE system (Classification of Recommendations, Evaluation, Development and Evaluations) [30,31]. Any disagreement was resolved by consensus or a third reviewer (VCO). According to the four-level GRADE system, the evidence may range from high to very low quality, with low levels indicating that future high-quality trials are likely to change estimated effects. In the current review, evidence began from high quality and was downgraded for each of the following issues: serious imprecision when analysed sample less than 400 [32]; serious risk of bias when more than 25% of the analysed participants are from trials with a high risk of bias (i.e., PEDRO scores less than 7 out of 10) [33]; and serious inconsistency when I^2^ > 50%, visual inspection of forest plots or when pooling was not possible [21]. We evaluated the publication bias using visual inspection of funnel plots and the Egger’s test adopting an α = 0.1 when data from at least ten trials were pooled in the same meta-analysis [20,34].

## 3. Results

**Figure 1:**
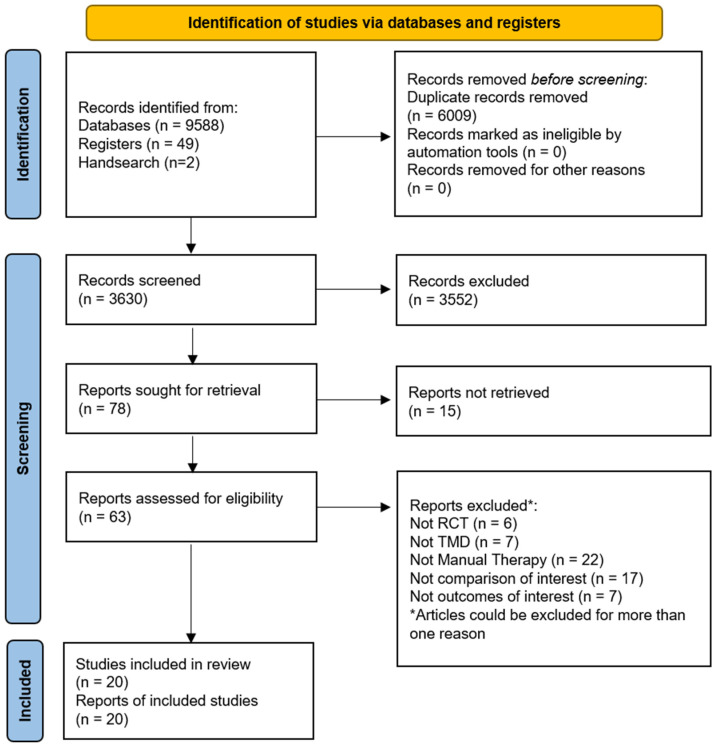
Flow of studies through the literature search and screening. RCT = randomised controlled trial; TMD = Temporomandibular disorders.

A total of 9639 records were retrieved from our searches, 6009 duplicates were removed, and the remaining 3630 titles and abstracts were screened. Then, 63 potential full texts were assessed for eligibility and 20 trials were included [35,36,37,38,39,40,41,42,43,44,45,46,47,48,49,50,51,52,53,54]. The study selection flow diagram is available in Figure 1.

### 3.1. Characteristics of Included Trials and Assessment of Risk of Bias

The included trials were published between 2005 and 2022, conducted in Spain (five trials), Brazil (four trials), Japan (two trials), USA (two trials), Turkey (two trials), Australia (one trial), Iran (one trial), Portugal (one trial), Thailand (one trial) and Croatia (one trial). Most of them (85%) used some version of the Diagnostic Criteria for TMD. Five trials investigated the effects of manual therapy versus control (sham or wait list) and fifteen trials investigated the additional effects of manual therapy on pain intensity (16 trials), MMO (16 trials) and/or disability (7 trials).

The modalities of manual therapy used were manual pressure release techniques (six trials), joint manipulation (four trials), joint mobilization (one trial), soft-tissue mobilization (one trial), stretching (one trial), instrumental-assisted techniques (two trials), massage (one trial), multimodalities (i.e., combination of two or more modalities of manual therapy) (five trials) and not specified (one trial). When outcome data was not adequately provided, we contacted the authors but received no answer, so we reported the findings that were available. Findings from trials with skewed data were reported separately. Further information regards the characteristics of the included trials are presented in Table 1.

The PEDRO scores of the included trials ranged from 1 to 9 points out of 10 (median = 7 points). Fourteen trials (70%) were classified as low risk of bias (i.e., scores ≥ 7 points). The main reasons for increasing risk of bias were not blinding therapists (20 trials [100%]), not blinding participants (14 trials [70%]), not performing concealed allocation (7 trials [35%]) and not blinding assessors and not performing an intention-to-treat analysis (5 trials [25%]). Detailed risk of bias assessment is presented in Table 2.

### 3.2. Effects of Manual Therapy on Pain Intensity in People with Temporomandibular Disorders

Four trials [39,42,44,45] investigated the effects of manual therapy when compared with control (sham or waiting list) and twelve trials investigated the additional effects of manual therapy when combined with other active intervention on pain intensity [35,36,38,41,43,46,47,49,50,51,52,53]. Seven trials used the 0–10 NRS [39,42,46,47,49,50,52], five trials used the 0–10 VAS [36,38,41,43,51] and four trials used the 0–100 VAS [35,44,45,53]. For pooling, outcome data were converted to a common 0–10 points scale.

#### 3.2.1. Manual Therapy versus Control on Pain Intensity

**Figure 2:**
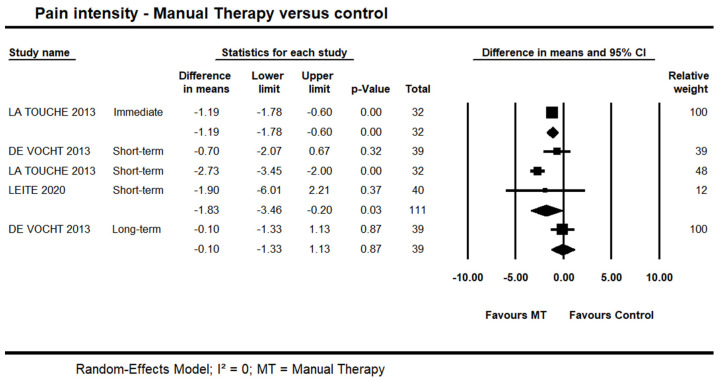
Forest plot of manual therapy versus control on pain intensity at immediate-, short- and long-term. Studies included were: Devocht et al., 2013 [39]; La Touche et al., 2013 [44]; Leite et al., 2020 [45].

One trial [44] provided low quality evidence of an immediate effect of manual therapy on pain intensity (MD = −0.88 points on the 0–10 points scale, 95% CI −1.57 to −0.19; n = 32). Data from three trials [39,44,45] also provided low quality evidence of a potential short-term effect for manual therapy on pain intensity (95% CI −3.46 to −0.20; I² = 0.0; n = 111). Long-term, one trial [39] provided very-low quality evidence of no difference (95% CI −1.33 to 1.13; n = 39) (Figure 2). It was not possible to include one trial in the pooling due to skewed data [42]. In this trial, the author reported a statistically significant difference in favour of manual therapy versus control at short- and long-terms; however, no detailed information in regards to the between-group difference and variability was reported.

#### 3.2.2. Additional Effects of Manual Therapy on Pain Intensity

Five trials [36,43,46,47,49] investigated the immediate additional effects of manual therapy on pain intensity, and two of them were not pooled due to skewed data [36] and lack of standard deviation measures [46]. Pooled data provided moderate quality evidence for no immediate additional effect of manual therapy on pain intensity (95% CI −1.61 to 0.10; I² = 0.0; n = 149). One of the two trials not pooled [36] also showed no immediate additional effect for manual therapy (*p* = 0.53); however, the other trial [46] suggested an additional effect of manual therapy combined with exercise when compared with exercises alone (−2.9 points out of 10).

Short-term, high-quality evidence from 10 trials showed an additional effect of manual therapy on pain intensity (95% CI −2.12 to −0.82; I² = 10; n = 434). Publication bias was not found (Supplementary File S3: Funnel plot), and sensitivity analysis removing trials with a high risk of bias [35,38] did not detect any potential impact on the estimates (95% CI −2.41 to −1.00; 8 trials, n = 394). We also conducted a subgroup analysis exploring whether different modalities of manual therapy impacted on the estimates; no impact was found. Findings are available on Supplementary File S4: Manual therapy modalities subgroup analysis on pain intensity.

**Figure 3:**
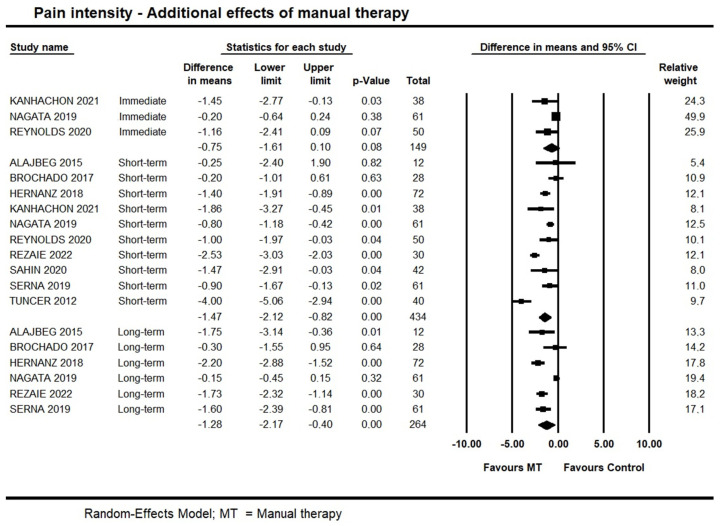
Forest plot of the additional effects of manual therapy on pain intensity at immediate-, short- and long-term. Studies included were: Alajbeg et al., 2015 [35]; Brochado et al., 2017 [38]; Hernanz et al., 2018 [41]; Kanhachon et al., 2021 [43]; Nagata et al., 2019 [47]; Reynolds et al., 2020 [49]; Rezaie et al., 2022 [50]; Sahin et al., 2020 [51]; Serna et al., 2019 [52]; Tuncer et al., 2012.

At long-term, moderate quality evidence from six trials supported an additional effect of manual therapy (95% CI −2.17 to −0.40; I² = 0.0; n = 342). Forest plots with estimates in the different time-points are shown in Figure 3.

### 3.3. Effects of Manual Therapy on Maximum Mouth Opening in People with Temporomandibular Disorders

Three trials [42,45,48] investigated the effects of manual therapy when compared with the control (sham or waiting list) and 13 trials investigated its additional effects on MMO [35,36,37,41,43,46,47,49,50,51,52,53,54]. MMO was assessed with Caliper in six trials [36,37,45,47,48,50], with Ruler in three trials [51,52,53], with other measurement tools in two trials [42,49] and four trials did not report the instrument used [35,41,46,54].

#### 3.3.1. Manual Therapy versus Control on Maximum Mouth Opening

**Figure 4:**
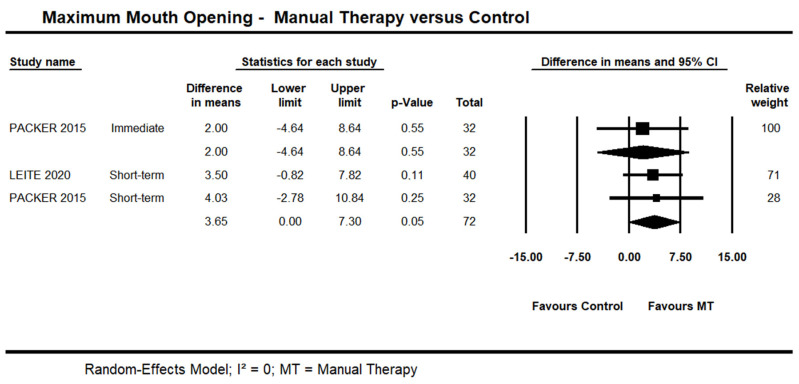
Forest plot of manual therapy versus control on maximum mouth opening at immediate- and short-term. Studies included were: Leite et al., 2020 [45]; Packer et al., 2015.

One trial [48] provided low quality evidence of no immediate effect of manual therapy on MMO (95% CI −4.64 to 8.64; n = 32). Short-term, moderate quality evidence from two trials [45,48] showed an effect on MMO (95% CI 0.01 to 7.30 mm; I² = 0.0; n = 72) (Figure 4). One trial [42] was not included in the pooling due to skewed data. A statistically significant difference was reported in favour of manual therapy versus control at the short- and long-term. No detailed information was available.

#### 3.3.2. Additional Effects of Manual Therapy on Maximum Mouth Opening

Seven trials [35,37,43,46,47,49,54] investigated the immediate additional effects of manual therapy on MMO. It was possible to pool data from five of them [35,37,43,47,49] due to a lack of standard deviations [46,54]. Moderate quality evidence demonstrated no between-group differences (95% CI −0.91 to 6.20 mm; I² = 0.0; n = 251). Contradictory findings were reported by the other two trials not included in the meta-analysis: Yoshida et al. [54] and Lucas et al. [46] found a between-group difference in favour of manual therapy on MMO.

Short-term, high-quality evidence from nine trials showed an additional effect of manual therapy on MMO (95% CI 1.58 to 3.58 mm; I² = 0; n = 494). Sensitivity analysis removing trials with high risk of bias [38] did not suggest an impact on the estimates (95% CI 1.43 to 3.88 mm; 8 trials, n = 394).

**Figure 5:**
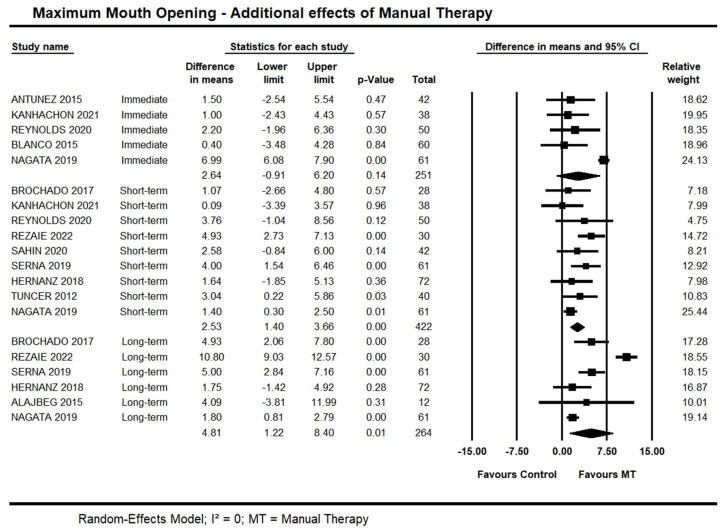
Forest plot of the additional effects of manual therapy on maximum mouth opening at immediate-, short- and long-term. Studies included were: Alajbeg et al., 2015 [35]; Antunez et al., 2015 [36]; Blanco et al., 2015 [37]; Brochado et al., 2017 [38]; Hernanz et al., 2018 [41]; Kanhachon et al., 2021 [43]; Nagata et al., 2019 [47]; Reynolds et al., 2020 [49]; Rezaie et al., 2022 [50]; Sahin et al., 2020 [51]; Serna et al., 2019 [52]; Tuncer et al., 2012 [53].

Long-term, moderate quality evidence from six trials showed an effect of manual therapy on MMO (95% CI 1.22 to 8.40 mm; I² = 0.0; n = 264). Sensitivity analysis removing trials with high risk of bias [35,38] did not suggest an impact on the estimates (95% CI 0.24 to 3.88; I² = 0.0; 4 trials, n = 224). Forest Plots with estimates at different time points are shown in Figure 5.

### 3.4. Effects of Manual Therapy on Disability in People with Temporomandibular Disorders

For disability, one trial [45] investigated the effects of manual therapy when compared with control (sham or wait list) and four trials investigated the additional effects of manual therapy [40,49,51,52]. The outcome measures used were the 0–68 MFIQ [45], the 0–100 Fonseca Patient History Index (FPHI) [40], the 0–8 points JFLS [51], the 0–20 points JFLS [49] and the 0–63 points Craniofacial Pain and Disability Inventory (CF-PDI) [52]. Due to the heterogeneity of measures, we reported SMD.

#### 3.4.1. Manual Therapy versus Control on Disability

Short-term, one trial [45] suggested an effect of manual therapy on disability when compared with control. Post-intervention disability differed 6.5% in favour of manual therapy (*p* = 0.01).

#### 3.4.2. Additional Effects of Manual Therapy on Disability

**Figure 6:**
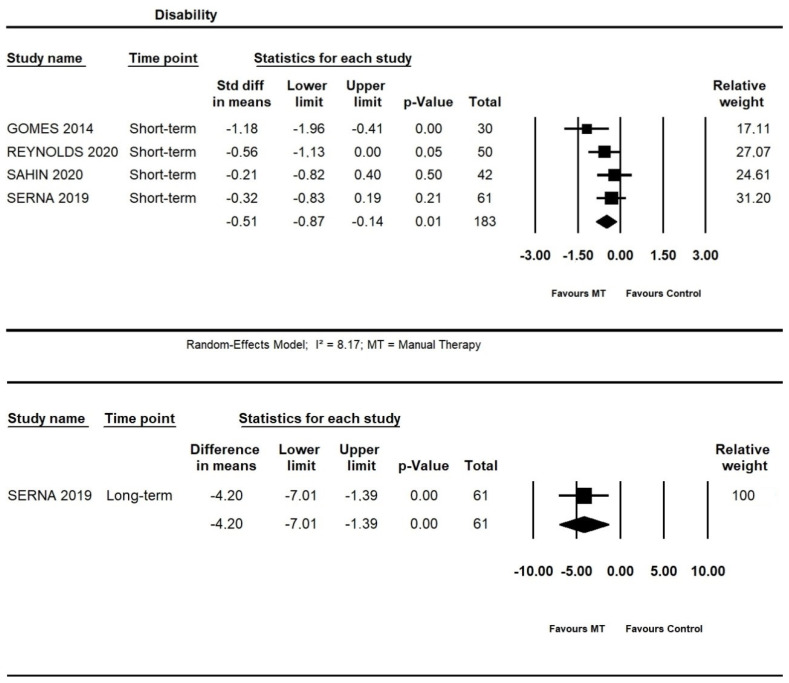
Forest plot of the additional effects of manual therapy on disability short- and long-term. The studies included were: Gomes et al., 2014 [40]; Reynolds et al., 2020 [49]; Sahin et al., 2020 [51]; Serna et al., 2019 [52].

At short-term, moderate quality evidence from four trials showed an additional effect of manual therapy on disability (SMD = −0.51, 95% CI −0.87 to −0.14; n = 183). At long-term, one trial [52] provided low quality evidence for an additional effect of manual therapy on the disability (95% CI −7.01 to −1.39; I² = 8.17; n = 61) (Figure 6).

The overall quality of evidence in the systematic review ranged from very low to high. The summary of findings with the GRADE assessment is reported in Table 3.

## 4. Discussion

This systematic review and meta-analysis found that manual therapy may have positive effects in the management of pain intensity, MMO and disability related to TMD; however, the effects’ sizes are small and may not be clinically relevant. Current quality of evidence ranged from very low to high, so future high quality RCTs are likely to change the estimates. Moderate quality of evidence supports joint manipulation, manual pressure, stretching and the combination of two or more manual therapies as additional therapies, with a similar small effect size. Therefore, the choice of the manual therapy technique should rely on the expertise of the health professional and preferences of the patient.

Our results corroborate with previous systematic reviews [17,19,55,56,57,58,59], which also found some positive effects in favour of manual therapy and other conservative interventions for pain intensity, MMO and disability, although the quality of the evidence has increased due to the inclusion of new trials. Among these, two recent systematic reviews [17,59] investigate the effects of conservative approaches in arthrogenic [17] and myogenic-related TMD [59]; however, manual therapy was considered as a general physical therapy approach and analysed together with other interventions such as exercises modalities, education and others. For that reason, our systematic review provides the most up-to-date evidence of manual therapy approaches for the management of TMD.

There are important issues to be addressed in order to improve the current state of the literature on this topic. The main reason to downgrade the level of evidence in our review was imprecision due to the sample size. Moreover, most of the included trials have a poor reporting quality and did not present data appropriately. Future high-quality RCTs should focus on recruit larger sample sizes and use the reporting checklist. Moreover, it is important to include economic evaluation and investigation of adverse events as outcomes to improve the decision-making process.

This systematic review was conducted with strong methodological rigor following recommendations. It updates and synthesizes all available evidence on the efficacy of manual therapy for pain intensity, MMO and disability in people with TMD. Estimating the effect sizes on critical outcomes for patients, assessing the certainty of evidence for each effect estimate and discussing the clinical relevance of the effect sizes across therapies informs patients and clinicians in their decision making. However, this review has some potential limitations. It included RCTs with patients with any diagnosis or type/classification (myogenic, arthrogenic or mixed) and also joint disorders. Subgroup analysis for the different classification of TMD was not possible due the limited number of trials including specifics types of TMD. Future trials should explore the effects of manual therapy in the different TMD diagnosis. In addition, our investigation was restricted to three clinical outcomes. It could be valuable to investigate other important clinical outcomes such as a health-related quality of life, pain pressure threshold and most importantly, the costs and adverse effects of the intervention.

## 5. Conclusions

We found moderate to high quality evidence of the positive effects of manual therapy modalities for pain intensity, maximum mouth opening and disability in temporomandibular disorders. However, the effect sizes are small and may not be clinically important. Future high-quality RCTs with larger sample sizes should explore the effects of manual therapy in the different TMD diagnosis, clarify adverse effects and include an economic evaluation for a better decision-making process.

## Figures and Tables

**Table 1:** Characteristics of the included trials (n = 20).

Study	Local	Participants	Intervention	Outcome Time-Points
Alajbeg et al., 2015 [35]	Croatia	12 participants (M = 3; F = 9), mean age of 30.5 ± 14 y/o, with TMJ disc displacement based on DC/TMD and MRI.	EG = Joint mobilization + Massage + Stabilization occlusal splint CG = Stabilization occlusal splint	Pain intensity (0–100 VAS); MMO Short and long-term.
Antunez et al., 2015 [36]	Spain	42 participants (M = 14; F = 28), mean age of 21.2 ± 1.6 y/o; TMD (myofascial pain) based on DC/TMD, for ≥6 months.	EG = Ischemic compression technique on the masseter muscle + stretching of hamstrings CG = PNF stretching of hamstrings	Pain intensity (0–10 VAS); MMO (Caliper); Immediate effects
Blanco et al., 2015 [37]	Spain	60 participants (M = 19; F = 41), mean age 35.2 ± 12 y/o, with TMD (myofascial pain) for ≥6 months based on DC/TMD; restricted cervical mobility.	EG = Suboccipital muscle inhibition + Pressure release massage + stretching. CG = Pressure release massage + stretching	MMO (Caliper); Immediate effects
Brochado et al., 2017 [38]	Brazil	28 participants (M = 1; F = 27), mean age 44.5 ± 17 y/o, with TMD (myogenic and arthrogenic) based on DC/TMD.	EG = Pressure Release Massage + Joint Mobilization + Photobiomodulation. CG = Photobiomodulation	Pain intensity (0–10 VAS) Short and long-term.
Devocht et al., 2013 [39]	USA	39 amateur athletes (M = 8; F = 31), mean age of 33 y/o, with TMD (myofascial pain) based on DC/TMD, for at ≥6 months.	EG = Mechanically assisted manipulation (hand-held spring-loaded instrument)—12 sessions for 2 months. CG = Sham Device	Pain intensity (0–10 NRS) Short and Long-term
Gomes et al., 2014 [40]	Brazil	30 participants (M = 4; F = 26), mean age of 27 ± 1.6 y/o, with severe TMD and bruxism.	EG = Massage + Occlusal splint—3 times week, for 4 weeks. CG = Occlusal splint	Oral Disability (0–100 FPHI) Short-term
Hernanz et al., 2018 [41]	Spain	72 participants (M = 12; F = 60), mean age 42 y/o, with TMD (myofascial pain) based on DC/TMD for ≥6 months.	EG = Pressure Release Technique + Occlusal splint + education CG = Sham + Occlusal splint and education	Pain intensity (0–10 VAS). MMO Short and long-term
Kalamir et al., 2011 [42]	Australia	60 participants (M = 26; F = 34), age between 18–50 y/o, with TMD based on DC/TMD for ≥3 months.	EG = Intraoral manual pressure—2 times week for 5 weeks CG = Waitlist	Pain intensity (0–10 NRS); MMO (caliper); Short and Long-term
Kanhachon et al., 2021 [43]	Thailand	38 academics (M = 4; F = 34), mean age of 25 ± 5 y/o, with pain on the neck, scapular, and jaw for more than 3 months, with a referral pattern.	EG = Active Stretching Release Therapy + hot pack on jaw and scapular areas + education CG = Hot pack on jaw and scapular areas + education	Pain intensity (0–10 VAS); MMO (therabite device)^®^ Immediate, short-term
La Touche et al., 2013 [44]	Spain	32 patients (M = 11; F = 21), mean age 34 y/o, with TMD (myofascial pain)—DC/TMD.	EG = Upper cervical mobilization—3 sessions over 2 weeks. CG = Sham	Pain intensity (0–100 VAS) Immediate, short-term
Leite et al., 2020 [45]	Brazil	48 women, age between 18–45 y/o, with TMD (pain dysfunction) based on DC/TMD, for ≥6 months.	EG = Diacutaneous Fibrolysis—2 sessions week for 4 weeks CG = Sham	Pain intensity (0–100 VAS); MMO (Calliper); Disability (0–68 MFIQ) Short-term
Lucas et al., 2017 [46]	Portugal	20 participants with pain on masticatory muscles and/or TMJ according to DC/TMD.	EG = Manual Therapy + Therapeutic Exercises—2 sessions week for 6 weeks CG = Therapeutic Exercises	Pain intensity (0–10 NRS); MMO Immediate effects
Nagata et al., 2019 [47]	Japan	61 participants (M = 11; F = 50), mean age of 49.6 ± 25 y/o, with TMD based on DC/TMD and MRI.	EG = Joint manipulation + self-exercise + CBT + education. CG = Self-exercise + CBT + education.	Pain intensity (0–10 NRS). MMO (caliper). Immediate, short and long-term
Packer et al., 2015 [48]	Brazil	32 women, mean age 24 ± 5 y/o, with TMD based on DC/TMD	EG = Upper thoracic manipulation CG = Sham	MMO (caliper). Immediate, short-term
Reynolds et al., 2020 [49]	USA	50 participants (M = 7; F = 43), mean age of 24.78 ± 5.4 y/o, with TMD according to DC/TMD.	EG = Cervical HVLAT + suboccipital release + education + home exercises CG = Sham HVLAT + suboccipital release + education + home exercises	Pain intensity (0–10 NRS); MMO (ROM scale). Disability (0–20 JFLS) Immediate, short-term
Rezaie et al., 2022 [50]	Iran	30 participants (M = 13; F = 17), mean age of 28 y/o, with TMD according to DC/TMD, for ≥3 months.	EG = Joint and soft-tissue mobilization on TMJ and cervical spine + Massage + UST + TENS CG: Massage + UST + TENS	Pain intensity (0–10 NRS); MMO (Calliper); Short and long-term
Sahin et al., 2020 [51]	Turkey	42 participants (M = 10; F = 32), mean age of 26.2 y/o, with TMD according to DC/TMD and trigger-point in the masseter muscle.	EG = Ischemic compression technique + Postural and Rocabado’s 6 × 6 exercises. CG =Postural and Rocabado’s 6 × 6 exercises	Pain intensity (0–10 VAS). MMO (Ruler). Disability (JFLS-8) Short-term
Serna et al., 2019 [52]	Spain	61 participants (M = 25; F = 36), age between 18 and 65 y/o, with tinnitus symptoms and TMD according to DC/TMD.	EG = Multimodal Manual therapy + Cervical and TMJ exercises + Self-massage + education—for 5 weeks CG = Cervical and TMJ exercises + Self-massage + education	Pain intensity (0–10 NRS); MMO (Adapted-Ruler); Disability (0–63 CF-PDI) Short and long-term
Tuncer et al., 2012 [53]	Turkey	40 participants (M = 9; F = 31), age between 18–72 y/o, with TMD and disc displacement based on DC/TMD for ≥3 months.	EG = Soft tissue and joint mobilization + TMJ exercises and stretching + Education CG = TMJ exercises and stretching + Education	Pain intensity (0–100 VAS); MMO (Ruler) Short-term
Yoshida et al., 2005 [54]	Japan	305 participants (M = 76; F = 229), age between 18–74 y/o, with TMJ disc displacement.	EG = Jaw joint manipulation + NSAIDs CG = NSAIDs	MMO Immediate effects

TMD = Temporomandibular disorder; TMJ = Temporomandibular Joint; M = Male; F = Female; y/o = years old; DC/TMD = Diagnostic Criteria Temporomandibular Disorders; VAS = Visual Analogue Scale; NRS = Numerical Rating Scale; MMO = Maximum Mouth Opening (with or without pain); EG = Experimental Group; CG = Control Group; CBT = Cognitive-behavioural therapy; PNF = Proprioceptive Neuromuscular Facilitation; HVLAT= High-velocity, low amplitude technique; FPHI = Fonseca Patient History Index; NDI = Neck disability Index; MFIQ = Migraine Functional Impact Questionnaire; JFLS = Jaw Functional Limitation Scale; MRI = Magnetic Resonance Imaging; CF-PDI = Craniofacial Pain and Disability Inventory; NSAID = Nonsteroidal anti-inflammatory drug.

**Table 2:** Risk of bias assessment—Pedro scale (n = 20).

Study	A	B	C	D	E	F	G	H	I	J	Score (0–10)
Alajbeg et al., 2015 [35]	Y	N	Y	N	N	Y	Y	Y	N	Y	6
Antunez et al., 2015 [36]	Y	N	Y	N	N	Y	Y	Y	N	Y	6
Blanco et al., 2015 [37]	Y	N	Y	Y	N	Y	Y	Y	Y	Y	8
Brochado et al., 2017 [38]	Y	N	Y	N	N	Y	N	Y	Y	Y	6
Devocht et al., 2013 [39]	Y	Y	Y	N	N	N	N	Y	Y	Y	6
Gomes et al., 2014 [40]	Y	Y	N	N	N	Y	Y	Y	Y	Y	7
Hernanz et al., 2018 [41]	Y	Y	Y	Y	N	N	Y	N	Y	Y	7
Kalamir et al., 2011 [42]	Y	Y	Y	N	N	Y	Y	Y	Y	N	7
Kanhachon et al., 2021 [43]	Y	Y	Y	N	N	Y	Y	Y	Y	Y	8
La Touche et al., 2013 [44]	Y	Y	Y	Y	N	Y	Y	Y	Y	Y	9
Leite et al., 2020 [45]	Y	N	Y	Y	N	Y	Y	Y	Y	Y	8
Lucas et al., 2017 [46]	Y	N	N	N	N	N	Y	N	N	N	2
Nagata et al., 2019 [47]	Y	Y	Y	N	N	N	Y	Y	Y	Y	7
Packer et al., 2015 [48]	Y	Y	Y	N	N	Y	Y	Y	N	Y	7
Reynolds et al., 2020 [49]	Y	Y	Y	Y	N	Y	Y	Y	Y	Y	9
Rezaie et al., 2022 [50]	Y	Y	Y	Y	N	Y	N	N	Y	Y	7
Sahin et al., 2020 [51]	Y	Y	Y	N	N	Y	Y	N	Y	Y	7
Serna et al., 2019 [52]	Y	Y	Y	N	N	Y	Y	Y	Y	Y	8
Tuncer et al., 2012 [53]	Y	Y	Y	N	N	Y	Y	Y	Y	Y	8
Yoshida et al., 2005 [54]	Y	N	N	N	N	N	N	N	N	N	1

Y = yes; N = no; A = Random allocation; B = Concealed allocation; C = Baseline Comparability; D = Blind subjects; E = Blind therapists; F = Blind assessors; G = Adequate follow-up; H = Intention-to-treat analysis; I = Between-group comparisons; J = Point estimates and variability.

**Table 3:** Summary of findings with grade assessment (n = 20).

**Population:** People with Temporomandibular Disorder. **Intervention:** Manual Pressure Release techniques (6 trials); Joint manipulation (4 trials); Joint mobilization (1 trial); Soft-tissue mobilization (1 trial); Stretching (1 trial); Instrumental-assisted techniques (2 trials); Massage (1 trial); MTs in combination (5 trials); Not specified (1 trial). **Comparison:** No intervention (13 trials), sham (6 trials), wait-list (1 trial). **Outcome:** Pain intensity (15 trials); MMO (15 trials); Disability (5 trials). **Setting:** Spain (5 trials); Brazil (4 trials); Japan (2 trials); USA (2 trials); Turkey (2 trials); Australia (1 trial); Iran (1 trial); Portugal (1 trial); Thailand (1 trial); Croatia (1 trial).				
**Outcome** **Time-Point**	**MD or SMD (CI 95%)**	**Sample Size** **(No. of Studies)**	**GRADE Assessment**	**Comments**
MT vs. Control 0–10 Pain intensity Immediate-effects	−0.88 (−1.57 to −0.19)	32 (1 study)	⨁⨁⊝⊝ LOW ^a,b^	The difference is statistically significant but not clinically important based on a MCID = 2.
MT add effects 0–10 Pain intensity Immediate-effects	−0.75 (−1.61 to 0.10)	149 (3 studies)	⨁⨁⨁⊝ MODERATE ^a^	The difference is not statistically significant.
MT vs. Control 0–10 Pain intensity Short-term	−1.83 (−3.46 to −0.20)	111 (3 studies)	⨁⨁⊝⊝ LOW ^a,c^	The difference is statistically significant but may not be clinically important based on a MCID = 2.
MT add effects 0–10 Pain intensity Short-term	−1.47 (−2.12 to −0.82)	434 (10 studies)	⨁⨁⨁⨁ HIGH	The difference is statistically significant but may not be clinically important based on a MCID = 2.
MT vs. Control 0–10 Pain intensity Long-term	−0.10 (−1.33 to 1.13)	39 (1 study)	⨁⊝⊝⊝ VERY LOW ^a,b,c^	The difference is not statistically significant.
MT add effects 0–10 Pain intensity Long-term	−1.28 (−2.17 to −0.40)	342 (6 studies)	⨁⨁⨁⊝ MODERATE ^a^	The difference is statistically significant but may not be clinically important based on a MCID = 2.
Joint Manipulation 0–10 Pain intensity Short-term	−0.83 (−1.18 to −0.47)	111 (2 studies)	⨁⨁⨁⊝ MODERATE ^a^	Subgroup analysis—MT modalities The difference is statistically significant but not clinically important based on a MCID = 2.
Manual Pressure 0–10 Pain intensity Short-term	−1.41 (−1.89 to −0.93)	114 (2 studies)	⨁⨁⨁⊝ MODERATE ^a^	Subgroup analysis—MT modalities The difference is statistically significant but not clinically important based on a MCID of 2 points.
Multimodal 0–10 Pain intensity Short-term	−1.65 (−2.98 to −0.32)	171 (5 studies)	⨁⨁⨁⊝ MODERATE ^a^	Subgroup analysis—MT modalities The difference is statistically significant but may not be clinically important based on a MCID = 2.
Stretching 0–10 Pain intensity Short-term	−1.86 (−3.27 to −0.45)	3 8(1 study)	⨁⨁⊝⊝ LOW ^a,b^	Subgroup analysis—MT modalities The difference is statistically significant but may not be clinically important based on a MCID = 2.
MT vs. Control MMO—mm Immediate-effects	2.0 (−4.64 to 8.64)	32 (1 study)	⨁⨁⊝⊝ LOW ^a,b^	The difference is not statistically significant.
MT add effects MMO—mm Immediate-effects	2.64 (−0.91 to 6.20)	251 (5 studies)	⨁⨁⨁⊝ MODERATE ^a^	The difference is not statistically significant.
MT vs. Control MMO—mm Short-term	3.65 (0.00 to 7.30)	72 (2 studies)	⨁⨁⨁⊝ MODERATE ^a^	The difference is statistically significant but may be not clinically important based on a MDC of 5 mm
MT add effects MMO—mm Short-term	2.5 8(1.58 to 3.58)	494 (9 studies)	⨁⨁⨁⨁ HIGH	The difference is statistically significant but not clinically important based on a MDC of 5 mm
MT add effects MMO—mm Long-term	4.81 (1.22 to 8.40)	264 (6 study)	⨁⨁⨁⊝ MODERATE ^a^	The difference is statistically significant but may not be clinically important based on a MDC = 5 mm.
MT add effects Disability Short-term	−0.51 (−0.87 to −0.14) *	183 (4 studies)	⨁⨁⨁⊝ MODERATE ^a^	The difference is statistically significant and may have a Moderate effect size based on the Hedges’g cut-off point of 0.5.
MT vs. Control Disability Long-term	−4.20 (−7.01 to −1.39)	61 (1 study)	⨁⨁⊝⊝ LOW ^a,b^	The difference is statistically significant but not clinically important based on a MDC = 8.
GRADE Working Group grades of evidence High certainty: We are very confident that the true effect lies close to that of the estimate of the effect; Moderate certainty: We are moderately confident in the effect estimate: The true effect is likely to be close to the estimate of the effect, but there is a possibility that it is substantially different; Low certainty: Confidence in the effect estimate is limited: The true effect may be substantially different from the estimated; Very low certainty: We have very little confidence in the effect estimate: The true effect is likely to be substantially different from the estimate of effect.				
Criteria for downgrade the certainty of evidence ^a^ Downgraded owing to imprecision: Sample size < 400; ^b^ Downgraded owing to inconsistence: When I² > 50% or when pooling was not possible; ^c^ Downgraded owing to risk of bias: >25% of the participants were from studies with a high risk of bias.				

MD = Mean difference; SMD = Standardized Mean Difference; * = SMD; MT = Manual Therapy; MT vs. Control = Comparison of manual therapy versus sham, placebo and wait-list; MT add effects = Comparison of manual therapy combined with other active intervention versus the active intervention alone; mm = millimetres; MCID = Minimum Clinically Important Difference; MDC = Minimal Detectable Change; MMO = Maximum Mouth Opening with or without pain; Instrumental MT = Use of devices to assist on manual therapy techniques; Manual Pressure = digital or manual pressure applied on a specific muscle area; Multimodal = Combination of two or more manual therapies.

## Supplementary Materials

The following supporting information can be downloaded at: https://www.mdpi.com/article/10.3390/life13020292/s1, Supplementary File S1: PRISMA Checklist; Supplementary File S2: Search Strategy; Supplementary File S3: Funnel plot; Supplementary File S4: Manual therapy modalities subgroup analysis on pain intensity.

## Abbreviations

**Abbreviation**	**Definition**
CF-PDI	Craniofacial pain and disability inventory
CIs	Confidence intervals
DC/TMD	The Diagnostic Criteria for Temporomandibular Disorders
FPHI	Fonseca Patient History Index
GRADE	Grading of Recommendations Assessment
JFLS	Jaw Functional Limitation Scale
MCID	Minimal clinical important difference
MDC	Minimal Detectable Change
MDs	Mean differences
MFIQ	Mandibular Function Impairment Questionnaire
MFIQ	Migraine Functional Impact Questionnaire
MMO	Maximum mouth opening
NRS	Numerical Rating Scale
PRISMA	Prospective Reporting Items for Systematic Review and Meta-Analyses
PROSPERO	Prospective Registry of Systematic Reviews
RCTs	Randomised controlled trials
SDs	Standard deviations
SMDs	Standardized mean differences
TMD	Temporomandibular disorders
VAS	Visual Analog Scale
