# Supplementary material for: The Efficacy of Manual Therapy Approaches on Pain, Maximum Mouth Opening and Disability in Temporomandibular Disorders: A Systematic Review of Randomised Controlled Trials
Source: Life (Basel). 2023 Jan 20;13(2):292. doi: 10.3390/life13020292 (PMC9967117; doi:10.3390/life13020292)
Supplement: Supplementary file 1 [file life-13-00292-s001.zip › Supplementary file S2 Search Strategy.pdf]

Supplementary material S2. Search strategy conducted on October 3rd 2022

OVID (MEDLINE, COCHRANE, EMBASE, AMED)

1. randomised controlled trial*.mp. [mp=ab, hw, ti, tn, ot, dm, mf, dv, kf, fx, dq, bt, nm, ox, px, rx, ui, sy, sh, kw, tx, ct]
2. Randomized Controlled Trial.mp. [mp=ab, hw, ti, tn, ot, dm, mf, dv, kf, fx, dq, bt, nm, ox, px, rx, ui, sy, sh, kw, tx, ct]
3. random allocation.mp. [mp=ab, hw, ti, tn, ot, dm, mf, dv, kf, fx, dq, bt, nm, ox, px, rx, ui, sy, sh, kw, tx, ct]
4. Comparative Stud*.mp. [mp=ab, hw, ti, tn, ot, dm, mf, dv, kf, fx, dq, bt, nm, ox, px, rx, ui, sy, sh, kw, tx, ct]
5. Controlled Clinical Trial*.mp. [mp=ab, hw, ti, tn, ot, dm, mf, dv, kf, fx, dq, bt, nm, ox, px, rx, ui, sy, sh, kw, tx, ct]
6. double-blind method*.mp. [mp=ab, hw, ti, tn, ot, dm, mf, dv, kf, fx, dq, bt, nm, ox, px, rx, ui, sy, sh, kw, tx, ct]
7. single-blind method*.mp. [mp=ab, hw, ti, tn, ot, dm, mf, dv, kf, fx, dq, bt, nm, ox, px, rx, ui, sy, sh, kw, tx, ct]
8. Clinical Trial*.mp. [mp=ab, hw, ti, tn, ot, dm, mf, dv, kf, fx, dq, bt, nm, ox, px, rx, ui, sy, sh, kw, tx, ct]
9. crossover stud*.mp. [mp=ab, hw, ti, tn, ot, dm, mf, dv, kf, fx, dq, bt, nm, ox, px, rx, ui, sy, sh, kw, tx, ct]
10. 1 or 2 or 3 or 4 or 5 or 6 or 7 or 8 or 9
11. temporomandibular.mp. [mp=ab, hw, kw, ti, ot, tn, dm, mf, dv, kf, fx, dq, bt, nm, ox, px, rx, an, ui, sy, sh, tx, ct]
12. temporomandibular disorder*.mp. [mp=ab, hw, kw, ti, ot, tn, dm, mf, dv, kf, fx, dq, bt, nm, ox, px, rx, an, ui, sy, sh, tx, ct]
13. 11 or 12
14. 10 and 13

PEDro

Abstract & Title: temporomandibular
Therapy: not applicable
Problem: not applicable
Body Part: head or neck
Subdiscipline: not applicable
Topic: not applicable
Method: not applicable
Author/Association: not applicable
Title Only: not applicable
Source: not applicable
Published Since: not applicable
New records added since: not applicable
Score of at least: not applicable
