# Supplementary material for: The Efficacy of Manual Therapy Approaches on Pain, Maximum Mouth Opening and Disability in Temporomandibular Disorders: A Systematic Review of Randomised Controlled Trials
Source: Life (Basel). 2023 Jan 20;13(2):292. doi: 10.3390/life13020292 (PMC9967117; doi:10.3390/life13020292)
Supplement: Supplementary file 1 [file life-13-00292-s001.zip › Supplementary file S3 Funnel Plot.pdf]

Supplementary file S3: Funnel Plot

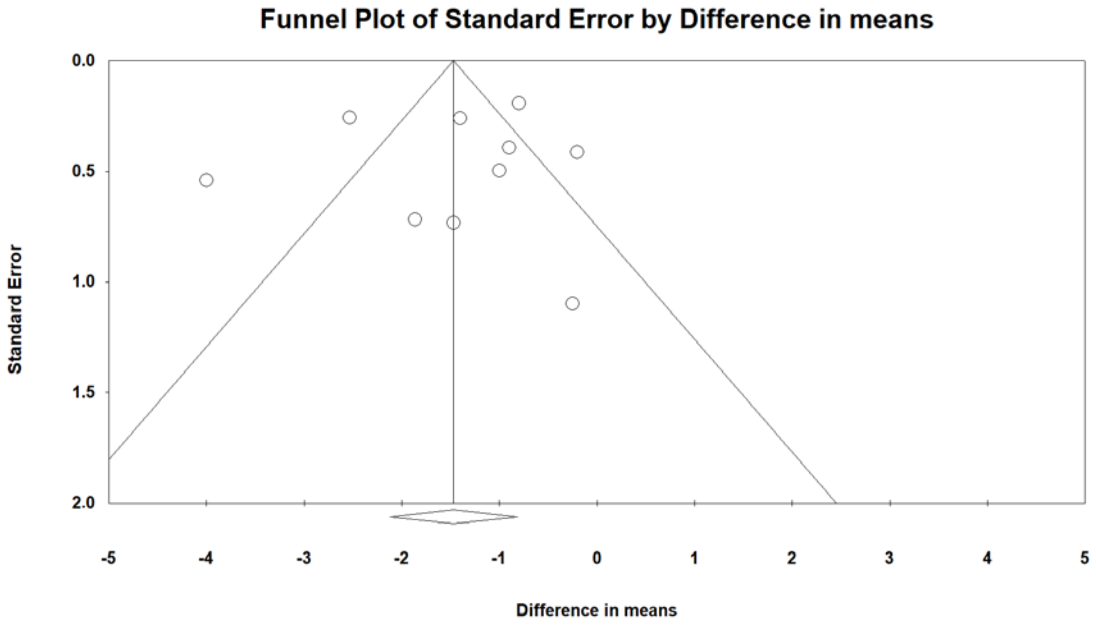

Egger's regression intercept
Intercept -0.69;
Standard error 1.81;
95% lower limit (2-tailed) -4.80;
95% upper limit (2-tailed) 3.41;
t-value 0.38; df 9.0;
P-value (1-tailed) 0.35;
P-value (2-tailed) 0.71.
