# Supplementary material for: The Efficacy of Manual Therapy Approaches on Pain, Maximum Mouth Opening and Disability in Temporomandibular Disorders: A Systematic Review of Randomised Controlled Trials
Source: Life (Basel). 2023 Jan 20;13(2):292. doi: 10.3390/life13020292 (PMC9967117; doi:10.3390/life13020292)
Supplement: Supplementary file 1 [file life-13-00292-s001.zip › Supplementary file S4 Manual therapy modalities subgroup analysis on pain intensity.pdf]

Supplementary file S4: Manual therapy modalities subgroup analysis on pain intensity

Pain intensity - Additional effects of manual therapy - Subgroup analysis

Study name	MT modality		Statistics for each study: Difference in means	Lower limit	Upper limit	p-Value	Total	Relative weight
NAGATA 2019	Joint manipulation	Short-term	-0.80	-1.18	-0.42	0.00	61	86
REYNOLDS 2020	Joint manipulation	Short-term	-1.00	-1.97	-0.03	0.04	50	13
	Joint manipulation		-0.83	-1.18	-0.47	0.00	111	
HERNANZ 2018	Manual pressure	Short-term	-1.40	-1.91	-0.89	0.00	72	88
SAHIN 2020	Manual pressure	Short-term	-1.47	-2.91	-0.03	0.04	42	11
	Manual pressure		-1.41	-1.89	-0.93	0.00	114	
ALAJBEG 2015	Multimodal	Short-term	-0.25	-2.40	1.90	0.82	12	14
BROCHADO 2017	Multimodal	Short-term	-0.20	-1.01	0.61	0.63	28	21
REZAIE 2022	Multimodal	Short-term	-2.53	-3.03	-2.03	0.00	30	22
SERNA 2019	Multimodal	Short-term	-0.90	-1.67	-0.13	0.02	61	21
TUNCER 2012	Multimodal	Short-term	-4.00	-5.06	-2.94	0.00	40	20
	Multimodal		-1.65	-2.98	-0.32	0.01	171	
KANHACHON 2021	Stretching	Short-term	-1.86	-3.27	-0.45	0.01	38	100
	Stretching		-1.86	-3.27	-0.45	0.01	38	

Difference in means and 95% CI

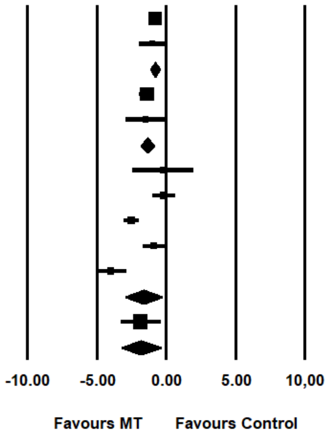

Random-Effects Model; MT = Manual Therapy
